# Patient-centred innovation to ensure access to diabetes care in Cambodia: the case of MoPoTsyo

**DOI:** 10.1186/s40545-016-0050-1

**Published:** 2016-01-21

**Authors:** Josefien van Olmen, Natalie Eggermont, Maurits van Pelt, Heang Hen, Jeroen de Man, François Schellevis, David H. Peters, Maryam Bigdeli

**Affiliations:** Department of Public Health Antwerp, Institute of Tropical Medicine, Nationalestraat 155, 2000 Antwerp, Belgium; Department of General Practice & Elderly Medicine, EMGO, Institute for Health and Care Research, VU University Medical Center, Van der Boechorststraat 7, 1081 Amsterdam, The Netherlands; University Hospital, University Hospital Brussels, Laarbeeklaan 101, Brussels, Belgium; MoPoTsyo, #9E, Street 3C, Phum Trea 1, Stung Meanchey, 12352 Phnom Penh Cambodia; NIVEL, Netherlands Institute for Health Services Research, Otterstraat 118-124, Utrecht, 3513 CR The Netherlands; Johns Hopkins Bloomberg School of Public Health, 615 N Wolfe St, Baltimore, MD 21205 USA; World Health Organisation, Alliance for Health Policy and Systems Research, 20 avenue Appia, 1211 Geneva, Switzerland

**Keywords:** Chronic diseases, Chronic conditions, Chronic care, Diabetes care, Patient-centred, Self management, Peer educator, Health systems research, Health system analysis, Stakeholder analysis

## Abstract

**Background:**

The increasing prevalence of chronic diseases puts a high burden on the health care systems of Low and Middle Income Countries which are often not adapted to provide the care needed. Peer support programmes are promoted to address health system constraints. This case study analyses a peer educator diabetes programme in Cambodia, MoPoTsyo, from a health system’s perspective. Which strategies were used and how did these strategies change? How is the programme perceived?

**Methods:**

Data were collected through semi-structured interviews with patients, MoPoTsyo staff and peer educators, contracted pharmacy staff and health workers, health care workers and non-contracted pharmacists and managers and policy makers at district, provincial and national level. Four areas were purposively selected to do the interviews. An inductive content analysis was done independently by two researchers.

**Results:**

MoPoTsyo developed into three stages: a focus on diabetes self-management; a widening scope to ensure affordable medicines and access to other health care services; and aiming for sustainability through more integration with the Cambodian public system and further upscaling. All respondents acknowledged the peer educators’ role and competence in patient education, but their ideas about additional tasks and their place in the system differed. Indirectly involved stakeholders and district managers emphasized the particular roles and responsibilities of all actors in the system and the particular role of the peer educator in the community. MoPoTsyo’s diagnostics and laboratory services were perceived as useful, especially by patients and project staff. Respondents were positive about the revolving drug fund, but expressed concerns about its integration into the government system. The degree of collaboration between health care staff and peer educators varied.

**Conclusion:**

MoPoTsyo responds to the needs of people with diabetes in Cambodia. Key success factors were: consistent focus on and involvement of the target group, backed up by a strong organisation; simultaneous reduction of other barriers to care; and the ongoing maintenance of relations at all levels within the health system. Despite resistance, MoPoTsyo has established a more balanced relationship between patients and health service providers, empowering patients to self-manage and access services that meet their needs.

## Background

The increasing prevalence of chronic diseases leads to large numbers of people in need of lifelong medical treatment, with diabetes mellitus providing one of the most striking examples [1–4]. The health care systems in many Low and Middle Income Countries (LMICs) are not well organized to deliver care for these diseases [1, 5, 6]. Medication policies do not cover the necessary essential drugs and many health facilities lack the knowledge, materials, and manpower for chronic disease management [7, 8]. People with chronic diseases receive episodic care, for which they often pay themselves in private sector facilities. The latter do not pay much attention to prevention and self-management.

Peer support programmes are among the innovations being promoted to address these health system constraints [9]. Linkage to clinical care is therefore identified as a key function [10]. However, most programs, and their evaluations, focus on overcoming the psychosocial, psychological and educational barriers to self-management and care [11–14]. Less is known about how these programs develop in and relate to the surrounding health care system.

This paper aims to contribute to the knowledge base in this field through an evaluative analysis of ‘MoPoTsyo’, a peer-educator programme for people with diabetes in Cambodia, from a health systems’ perspective. The analysis takes into account essential context elements, the dynamic nature of the implementation, and the perspectives of direct and indirect stakeholders [15–17]. We address the following questions: 1) Which strategies were used by the organisation to reach its goals and how did these strategies change over time to adapt to their context? 2) How is the programme perceived by stakeholders of the wider health system?

The insights from this paper might be useful for organizations in other LMICs improving care programmes for people with chronic diseases in which peers are involved.

## Methods

This instrumental case study aims to analyse in-depth a particular example of a patient-centred programme within the overall health system, and draw lessons for other patient-based initiatives [18]. This case was purposively selected based upon the content of the programme, the innovative approach used to ensure access to medication, the context and the access to informants and data for analysis.

### Context of the Cambodian health care system

The national estimated prevalence of diabetes mellitus is 3.0 % [19]. The Cambodian society and its health care system show traces of a long period of war from the 1970’s to the 1990’s, followed by a post-war reconstruction phase with massive influx of foreign development aid and rapid economic development. Although development aid has decreased drastically in recent years, the government health budget continues to be complemented by external resources. The Human Development Index is 0.543, life expectancy at birth 63.6 years and the GNI 2095 PPP$ per capita [20]. The Cambodian health care system is mixed. In the public health services, care for people with diabetes is hampered through a lack of medicines and training of staff [21]. Studies report that more than half of patients with diabetes in Cambodia remained untreated [22]. In practice, care outside of the national hospitals in the capital and the provincial hospitals is limited. Some private-not-for-profit hospitals have launched diabetes care projects, but these proved difficult to sustain without external support [23, 24]. In urban areas, the private health sector has grown steadily and many small-scale health service providers, especially lower echelon providers, get most of their revenues through selling pharmaceuticals. Diabetes is costly to patients and their families. Diabetes-related complications, such as renal failure, are typically already highly prevalent at the time of diagnosis: a retrospective analysis from 483 people screened showed that among those diagnosed with diabetes mellitus, 57.5 % had an estimated glomerular filtration rate below 60 ml/min/1.73 m2 [25].

### The case

MoPoTsyo is a Cambodian Non-Governmental Organization (NGO) established in 2004. Its aim is to empower people with diabetes to self-manage their condition by creating networks of community-based diabetes peer educators who share their knowledge with other patients. Data about outcomes of the programme have been published elsewhere. In a study cohort of 484 patients, the male/female ratio was 0/40 and the median age 55 years (49–62). 27.4 % of the patients were illiterate and 30.4 % had only primary education. Median HbA1C was 54.1 mmol/mol. Two third of the patients monitored their glucose levels, mostly through urine test strips. Most people (84 %) were on Oral Diabetic Agents and they reported high levels of adherence [26]. An external evaluation in 2011 among 150 randomly selected patients, who had been in the programme for at least 2 years, showed similar results. There were improvements in fasting blood glucose levels and blood pressure from baseline, with about one third of patients reaching treatment targets for fasting blood glucose and two thirds for blood pressure levels. Patients indicated that MoPoTsyo helped them to address different barriers to care. They mentioned that participation increased self-efficacy, and improved access to care and medicines [27].

### Data collection and analysis

**Fig. 1:**
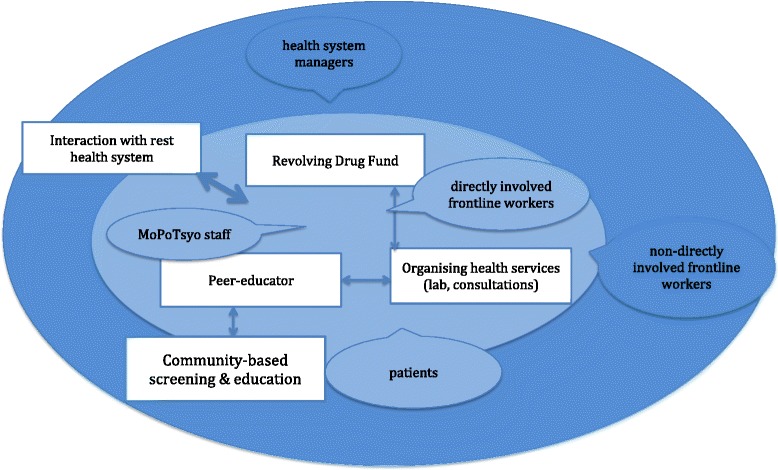
The conceptual approach for the analysis of the MoPoTsyo function as perceived by health system stakeholders

To understand how the programme is perceived within the health care system, we collected data through semi-structured interviews with stakeholders directly or indirectly involved in the programme, in order to explore their experiences and views of the programme. Two operational districts, each in a different province, were selected, for data collection. Selection of these areas was based upon the duration of the programme (existing for at least three years) and the variation in implementation and access to the terrain. In each district, key informants were selected. Interviewees from the following categories were contacted: a) MoPoTsyo staff and peer educators (8 interviewees); b) directly involved stakeholders, i.e. contracted pharmacy staff and health workers (3 interviewees); c) indirectly involved stakeholders at the frontline, i.e. health care workers and non-contracted pharmacists (10 interviewees); and d) managers and policy makers at district, provincial and national level (6 interviewees). Inclusion criteria for selection were: their position in the health system, their availability and their familiarity with the programme. Apart from the central level policy makers and MoPoTsyo staff, interviewees had to be working in the health system in the operational district. Further selection was done with the aim to get a wide array of different opinions, from both genders, different ages and towns as well as remote areas. Because of the relatively small study population, choices were limited. They were contacted by telephone, by a MoPoTsyo staff member. Apart from the group of policy makers at national level, where 1 out of the 3 persons agreed to be interviewed, all selected persons in the other categories accepted the invitation. Most interviewees were men of senior age. One female peer educator was interviewed. Themes followed from the strategy analysis and research questions and included: 1) the role of the peer educators in the community and within the health care system; 2) the Revolving Drug Fund (RDF); 3) MoPoTsyo’s role in organising health services; and 4) collaboration with and integration into the national health system. We show the conceptual approach used for the analysis in Fig. 1. The interview guides were tailored to each type of stakeholder. The interviews were done in 2014 as part of an additional evaluation of the (perceived) effects of MoPoTsyo in the health care system [28]. The interviews were audio recorded, verbatim transcribed, translated from Khmer into English and inductive content analysis was done independently by two researchers (NE, JVO). They independently marked and categorised key phrases and text fragments, starting from the 4 main themes, then looking for emerging themes [29]. Sources were triangulated: after the identification of themes, we studied project documents and additional literature and we gained more information from project managers and independent observers, looking for both consistency and different opinions. Where necessary, we reported on the differences in understanding of data. The case-study draft was reviewed by three key informants to discuss the interpretation of data.

Ethical approval for this study was obtained by all relevant authorities (ISRCTN 86247213).

## Results

### Evolution of the programme

MoPoTsyo comprised three stages: 1) Initially the programme focused on diabetes self-management education and community screening; 2) The programme then widened its scope to ensure access to medicines and diabetes related health care services; 3) It now aims for sustainability through integration of the peer educator network strategy with the Cambodian public health care system and further upscaling.

### Stage 1. Focus on diabetes self-management education and community screening

MoPoTsyo was established in 2004 to empower people living with diabetes to self-manage their condition by creating networks of community-based diabetes peer educators. A major driver was the observation of a poor exchange of information and communication between patients and health care providers. The observed ‘ *mismatch between what patients wanted to know and what they were allowed to ask and get in terms of information*’ was striking [30]. Especially for people with chronic diseases such as diabetes, providing adequate information can contribute to better (self-) management, which may in turn decrease the development of complications and the need for more specialized care, keeping costs affordable [31]. The initial aim of the programme was therefore to provide access to information for people with diabetes.

The programme trains people with diabetes in self-management and it also teaches them how to become a peer educator. Candidates are identified based upon their motivation, literacy level and social skills. The 6-week training curriculum (in Khmer) was developed by doctors, pharmacists and experienced peer educators and trains candidates to self-manage their disease. After the exam, they return to their own community to form new patient groups through active community screening, going to people’s houses, providing education on diabetes and offering to have their urine checked. They are linked with other peer educators and MoPoTsyo staff within the health district forming a network, through which reporting, supervision, continuous education, monitoring and evaluation can be organized. The number of diabetic patients per peer educator varies from 20 to 100, with most covering around 60 patients.

Peer educators receive basic equipment and supplies (e.g. a handheld glucometer and blood and urine glucose strips), and host weekly patient gatherings and education sessions in their homes, which act as patient information centres. Their activities (on average three half days per week) focus on providing people with diabetes with reliable information on nutrition and exercise and teaching them basic skills such as self-measurement of blood glucose levels, blood pressure and bodyweight. The peer educators are trained to perform blood glucose tests and general follow-up. In case a patient does not show up for follow-up, the peer educator will visit the patient at home to motivate him/her to continue treatment. The educators receive a travel cost reimbursement and financial incentives for service and performance, including incentives for activities such as screening, monitoring, and patient gatherings. On average , the monthly incentive adds up to USD 40.

### Stage 2a. Widening the scope through improving access to medicines via a revolving drug fund

At the start of the programme in a Phnom Penh urban slum, the peer educators’ activities complemented those of an international NGO project. The purpose of this project was to set up a subsidized diabetes clinic within a tertiary care National Hospital setting where patients could go for consultations, laboratory testing and medication. When donor funding for the international NGO project ended, prices of medicines and services in the clinic started to rise as the staff suddenly had to cover the full costs of the products and services it had been providing during the project.

Normally, the Ministry of Health supplies diabetes medication to the provincial hospitals to run their diabetes clinics and stipulates the regulations for hospital financing. Patients pay a user fee of which 39 % is meant to cover the clinic’s cost for providing the care including the cost of procuring extra medication in case government supplies are insufficient to cover the clinic’s needs. In practice, this is usually not sufficient to cover the costs. To raise more revenues, clinics increase the frequency of medical consultations, shorten the duration of the consultations, and dispense medicines for a shorter period of time. This makes care unaffordable for most patients, in particular for the ones who must cover larger distances.

MoPoTsyo’s management realized that, if they wanted to improve access to care for diabetes patients in this challenging context, they would need to go beyond just informing and educating patients. A Revolving Drug Fund (RDF) was thus set up. They established contracts with local pharmacies to sell selected generic medicines to MoPoTsyo members at a fixed price per tablet, to be paid out-of-pocket. MoPoTsyo procures medicines in bulk on the international market and sells them to the contracted pharmacies allowing them a 5–15 % profit margin, depending on the type of medicine. Every contracted pharmacy receives from the NGO a fridge and a special cupboard for the RDF medicines on loan. MoPoTsyo provides monthly supplies because most pharmacies do not have the required air-conditioned storage conditions. Regulations are in place and actively monitored to prevent fraudulent acts [32]. Comparing the medication prescribed and dispensed through an integrated database allows MoPoTsyo to monitor adherence. Pharmacies are selected in collaboration with the district health authorities, looking at proximity, trust and reputation. Until 2012, all were private pharmacies, but upon request of the Ministry, they started working with pharmacy outlets within public facilities. The RDF is an essential component of the MoPoTsyo service package, since it ensures availability of quality (generic) medicines to patients in remote districts. It has also become an important strategic asset for the MoPoTsyo organization. The revenue of the RDF is used to sustain the supply of medicines, collect the related data, pay public and private pharmacies for their performance, and finance part of the organization itself where donor funding falls short.

### Stage 2b. Widening scope through organising other health services

When MoPoTsyo started to pilot its activities in rural areas, another gap in the health system became evident. There were no consultation services for diabetes patients at health centres or district referral hospitals, due to a lack of capacity and motivation of local doctors. MoPoTsyo decided to contract experienced doctors from diabetes clinics in Phnom Penh to travel to district hospitals to carry out the diabetes consultations and build local capacity through training. The hospital directors were asked to appoint a local doctor to join the outreach consultation sessions (which are organized on a weekly or monthly basis) who was trained by the visiting consultant. During the consultations, peer educators play an active role in patient registration, blood pressure and blood glucose measurement, weighing, counselling and other tasks.

Patients are prescribed their medication by a medical doctor, who writes in the MoPoTsyo self-management book of the patient which medicines have to be taken when and how many. With this prescription, patients can then go to a contracted pharmacy, buy medication for a 3 month period (maximum) and get a refill after that. Patients go to see the doctor once to twice a year on average. Peer educators are supposed to monitor patients in the meantime and recommend earlier consultation if they observe that the medication is not adequate or on other indications [32].

In addition, MoPoTsyo started to develop its own capacity to carry out laboratory tests. At the referral hospitals these tests were either unavailable or unaffordable. Blood sample collection is carried out at the local health centre and serum-specimens are transported to the central lab. Prices charged to the members remain below those in other facilities. The laboratory tests are organized before the medical consultations, so the doctor can take into account the results. Other services, like diabetic retinopathy screening, are organized in collaboration with external (private not for profit) clinics.

Further widening its scope to other chronic conditions, MoPoTsyo also began to organize peer-support groups for non-diabetic people with hypertension.

MoPoTsyo performs an assessment of all areas where a peer educator is active, according to the Lot Quality Assurance System. This is a method to provide management information, using small number samples (less than 20), to identify, for instance, if areas reach the benchmark for vaccination coverage [33]. The assessments are performed by peer-educators from another province and the results are translated into a score per peer educator that indicates the quality of their work, which is then adjusted to the workload of the educator. The assessment results are used by the local supervisor and the programme management to address weaknesses and they are translated into a bonus reward for the educators that varies between USD 10 and USD 200. MoPoTsyo also organizes annual standardized patient satisfaction interviews about the pharmacy services as part of performance evaluations.

### Stage 3. Aiming for sustainability through integration and scaling up

MoPoTsyo has several sources of revenue. Donor funding is mostly used to set up a new network in a district where there was no prior activity. MoPoTsyo aims for a system that is financially sustainable (once it is well-established) with the RDF generating 80 % of the revenue. The incentives for the peer educators are mostly paid from patients’ contributions when they access their services. To ensure access to care for the poorest, the organization also ran a health equity fund for 2.5 years, which provided large discounts on the price of the routine medication for the most vulnerable diabetics using a voucher system. This system ended at the end of 2013 due to a lack of funds. The unit cost calculation of one diabetes patient receiving care in 2011 was USD 43.47 USD per year [32].

MoPoTsyo saw a steady growth of the number of involved districts and the number of people with diabetes or hypertension who registered as a member. At the end of 2014, 21,666 patients were registered, with 12,595 diabetic and 9071 non-diabetic hypertensive patients. The organization had 162 peer educators and 29 salaried staff members (one third suffering themselves from an NCD, mostly diabetes) [32].

The services were initially organized in a rather vertical way, with little involvement from the Cambodian public health system. MoPoTsyo increasingly tried to involve local authorities and public health facilities, for instance by organizing the medical consultations inside the public referral hospitals (2007), or later through sharing monitoring and evaluation data and analyses with district managers and the Ministry of Health. When setting up a network in a new area, now a link is immediately established with the public health services through collaboration with the pharmacies in the district hospital and organization of blood samples collection at the health centre for the laboratory testing.

In 2013, the Ministry of Health included the continuation and expansion of the Peer Educator Networks into its National Strategic Plan for Prevention and Control of NCDs 2013–2020. It took the decision to integrate the peer educator networks under the District health authorities as part of the formal health care system and allocated funds for management of the peer educator networks in 8 districts for 2013 and 2014 [34]. The details of the transition and the integration of the other services of MoPoTsyo into the system were the subjects of negotiation. At the time our data-collection was undertaken, the strategy of the MOH about how the transition would be implemented was still being debated and MoPoTsyo was still in charge of the peer educator networks, the RDF, the laboratory services and medical consultations in all districts where it was operational. In 2015, MoPoTsyo handed over the responsibility for the peer educators in a limited number of districts. They were linked with the primary care facilities and received a small salary from the district. The national funding strategy for further funding of this process is not yet clear.

### Perceptions on the MoPoTsyo programme

Views are presented here using the following themes: perception of the role of the peer educators; ofMoPoTsyo’s role and the role of peer educators in organizing health care services and the RDF in particular; of the integration of MoPoTsyo’s strategies within the public health care system. Table 1 gives a summary of the results of the in-depth interviews along the main themes. We present the views of the different subgroups of stakeholders: MoPoTsyo staff and educators, contracted pharmacy staff, non-contracted pharmacy staff, health care workers and health system managers.

**Table 1:** Summary of results of in depth interviews

	Categorisation	MoPoTsyo staff & peer educators	Directly involved frontline workers (pharmacists, health care workers contracted by MoPoTsyo)	Indirectly involved frontline workers (non-contracted health care workers, drug vendors)	Health system managers
On the role of peer-educators					
	Essential tasks and competences	Patient education	Patient education	Patient education	Patient education
		Variety in competence, depending on experience		Case detection, outreach activities	focus on diabetes and lifestyle expertise, retention in care
				Credibility in community	Place in the community
	Additional tasks	To be extended (with training)		Within limits (no treatment)	Very limited
		Patients’ demand	Patients’ demand	Patients’ demand	
	Formal Responsibilities	Need permission for extension of tasks			Clear dinstinction of responsibilities
	Risks				Individual peer educators malbehaving, (lack of) training
On RDF					
	Benefits	Core component	Good prices	Complementary to their own services (different customers)	Good quality, low cost, proximity
			Increased competence		
			Increased profits		
	Problems	Distance is a barrier for patients	Strict regulations, administrative burden		
		Uncertainty about sustainability			Different cost recovery system from public services
On MoPoTsyo’s role in organizing health care services					
	Benefits	Complementary to insufficient and/or expensive health services	Capacity development	Useful for case detection	Renders new patients to health services
		Proximity		Cheap alternative for some patients, temporary solution	Relieves burden of public system
	Position towards other health providers	Feeling of distrust from other health workers	Complementary	Complementary (different customers)	
On collaboration /integration into the health system					
	Exchange	On personal basis, few formal communication channels		On personal basis	On personal basis
	Plans for intergration	Uncertainty about management peers and RDF	Uncertainty, fear for loss of customers		uncertainty about financing and RDF managament
	Future role MoPoTsyo	Advocacy for stronger peer position		Support to health facilities	Capacity development

*PE* peer educator, *PEN* peer educator network

### Perceptions of the role of peer educators

The role of the peer educator has changed since the initiation of the programme. From being screeners, educators and activators of patients, some have turned into experts on which patients rely for their diabetes management [27].

Both directly and indirectly involved stakeholders at the frontline and the managers and policy makers regarded patient education as the essential task of peer educators and recognized their competence in doing so. (*“Peers are really important […] because they are diabetes patients, it’s easy for them to communicate with each other” –* a non-involved health worker). Their credibility in the community was recognised ( *“(…) most people believe him more than the health centre*” – a non-contracted health worker). Indirectly involved stakeholders and policy makers stressed the importance of peer educators for community-outreach, case-detection and health promotion, considering the model an improvement in efficiency, similar to the deployment of community health workers for other disease programs (*“We cannot go to the community but peers can do it”* - a district health manager *;* “*Peer […] are the eyes and nose in the community.” –* a non-involved health worker).

Opinions on additional tasks and their place in the system differed. Whereas all respondents confirmed that peer educators take up some additional tasks (blood glucose checks, blood pressure measurements), MoPoTsyo staff considered their tasks to include guidance of patients in their daily management, such as assistance during emergencies and subsequent changes in medication. The health workers not directly involved in MoPoTsyo and the managers and policy makers expressed a clear separation of responsibilities (“*When there is a hypo, we should tell patients to reduce drug intake*” – MoPoTsyo staff ; “*peers cannot treat patients*” – a non-contracted health worker; “*peers do their job by doctor’s explanation”-* district manager). The extent to which peers felt confident to address disease management issues seemed to depend on their expertise: “*I think it is no problem because I have experience and dealt about 100 times with this problem*.” versus “*I know little*, *as I only check and I cannot do more than that*” (two different educators). Some peer educators felt that they can take up more responsibilities, given appropriate training. Most patients considered the assistance of the peer educator essential, including diabetes management tasks, such as monitoring of glycaemia and self-injection of insulin. Non-contracted health care workers and district managers considered peer educator as community based workers, comparable with the role community health workers play for other diseases. They emphasized the particular roles and responsibilities of all actors in the system.

Doctors noted that peer educators lack the expertise to provide sufficient backgroundand broader interpretation of patients’ complaints which are not always linked to diabetes. This can delay (necessary) referral. Educators are aware of the limitations of their function, but they also mention that patients consider them as experts (even as ‘doctors’) and expect them to take up different tasks (“*They don’t know I am a patient, they know only I am a doctor.” “No one permits us to do that but patients demand that.”* – 2 different peer educators*).* Some district authorities expressed their concern about critical incidents that were reported about the behaviour of some peer educators and stressed the importance of support to peers by MoPoTsyo’s organisation *(“we worry about peers who get little support from the organization”*).

### Views on the organizing of health services by MoPoTsyo

MoPoTsyo staff members considered the extension of services as complementary to non-adequate or too expensive health services by other actors, for which there is a demand. (*“At the hospital, the doctor doesn’t have equipment. They never test blood”; “the hospital in present days doesn’t have medicine” ;“It isn’t a competition. They* [doctors in government services] *don’t know clearly.”* ; “[…] *every time she goes to [*hospital*], she has to pay about USD 150.”)* Peer educators felt rejected by other non-involved staff (*“The staff rarely asks and talks to us”;* “*Many other doctors also treat and sell medicine, for that reason they are not happy with us*”).

Other frontline health workers, both those directly working with MoPoTsyo and those further away, considered the peer services useful. They did not seem to consider MoPoTsyo’s organisation of laboratory services and medical consultations as significant. If they mentioned it, they described it as a cheap alternative for some patients or as a temporary solution. Neither of the groups considered this as competition. “*There was no care before, so the network is filling a gap, not stealing patients”* (front-line health worker).

District authorities recognised the expertise of MoPoTsyo in providing advice on diabetes, lifestyle changes and life-long retention in care (“*its focus on diabetes is mostly more than what is provided by my hospital services. […] lifestyle advice is more related to patients, better than at my place”)*. They also viewed the MoPoTsyo solutions as temporary, to overcome gaps in the government health system (“[…] *good that she can do it, because there aren’t many health resources in public [services]”)*. At the same time, both public and private doctors valued the tracing of patients in the community, who could then be brought to the public system (“[…] *people came to receive service at hospital, about 90 % of whom came via the peer of MoPoTsyo”; “they are helping us to advertise the service of our hospital as well” – government doctor;* “[I] *get a lot customers*”-private doctor).

### Perceptions of the RDF

MoPoTsyo staff considered poor access to drugs as a central problem for most patients and the RDF as an essential part of the solution. Peer educators pleaded for additional measures to further reduce barriers. Some peers organized “sharing services” to reduce transport costs. *(“Some people who live very far from our place, they buy drug from me [..] I can share my drug”)* (“*I could go to the house of the peer educator to get medicine.“ -* patient*).*

Contracted pharmacy staff were generally positive about the RDF. The profit on the sale of diabetes medication was relatively small, but they perceived an added benefit from the sale of other medicines and the stories patients told in the village about the positive effects of the drugs. *(“The profit from selling MoPoTsyo drug is little, but I can receive profit by selling other drugs.”)* They appreciated the prices against which they purchase the stock, but some complained about the logistical arrangements and administrative burden. One pharmacist mentioned increased competence as a benefit (“*after I have learnt about the drug […], I feel that it is not so difficult”)*. Non-contracted pharmacy staff did not perceive the contracted pharmacies as a threat. Most of them did not sell diabetes medicines and felt they served a different part of the market (“*They sell drug for a cheap price”)*. Lower-qualified drug-sellers said they were afraid to sell diabetes medication, because of their lack of knowledge (*“We don’t do this job, because we aren’t good at diabetes and hypertension disease.”)*.

Many decentralized health systems managers felt that *MoPoTsyo* offers good quality medicines close to patients’ homes at an affordable price (“*It is not far away, it is not so expensive and we can keep quality of drug*” *“Especially I can let poor patients have ability to use this service”* ). Central level authorities were wary of the RDF and the other services MoPoTsyo started to organize. They emphasized that the government is responsible for laboratory services, consultations and medicine supply. (“*Patients are there to receive medicine, not to organize care*” – central government representative). All respondents saw considerable problems to integrate the RDF into the government system. (“*We* [state] *offer drugs and consultation for a total price.[…] MoPoTsyo offers consultation and selling of drugs separately. […]* W*e worry about the management of drugs, we don’t know who will supply. Are there enough drugs for us? –* district health manager; “I*f MoPoTsyo services are transferred to the state to be managed […. ] we worry that the drugs will become more expensive or that we don’t have enough drugs to offer patients.”-* peer educator).

### Perceptions of the collaboration with and integration into the national health system

From the answers of all stakeholders it appears that exchange and collaboration are not institutionalised, but happens on an ad hoc and personal basis. Peer educator quotes were: *“We don’t have communication with the health support committee or commune”,* but also, *“He [subdirector of hospital] always comes to meet me to discuss about this thing*.” District manager quotes were similar: “*MoPoTsyo and I are sharing information each other”* “*we transfer them to him* [educator]” *“Cooperation between the health centre and many local [actors] does not exist yet.”*). This explains the differences in perception across health centre areas. In one health centre area, the peer educators had played an active role in outreach and they were in touch with all stakeholders. Front-line health workers from this area reported referring each diabetes patient to the peer educators and considered this as a form of cooperation beneficial to both parties. In another area where peer educators were less active, other health workers were not aware of their presence and new diabetes patients were referred to the district hospital.

The government stakeholders considered the weak collaboration with the public health system as a problem. At central level, they expressed the wish for MoPoTsyo to work together with the public health system, to strengthen the system overall. “*I think that MoPoTsyo has created a good system. But the bad thing is that it never used the existing public health system. And then when they finish, everything will be gone*.” (policy maker central level). They hoped MoPoTsyo could remain active in building capacity. *(“I want MoPoTsyo to train us at all levels”)*.

Stakeholders displayed uncertainty about the consequences of the integration of the peer educator network and other MoPoTsyo activities into the district health systems. Peer educators expressed concern about what was to happen when the district authorities would take over peer educator network management, as envisaged in the plans. “*I am waiting to see their policy and how they will manage us.” “I feel worried.”* Contracted pharmacies said they fear the end of the RDF *(“I think I will lose most customers.”).* District managers were not yet clear on how they would manage peer networks (“[…] *how to manage the peers if we don’t have budget?*”).

The different actors voiced different visions about diabetes care in Cambodia in the future. MoPoTsyo staff advocated for stronger relations with national and international partners, to become a facilitator of chronic care service delivery (“*peers should create relationships with national and international organizations”*). Stakeholders further away from MoPoTsyo saw the networks as a transition phase towards a more clinic-based model. For them, peer educators are beneficial in a resource constrained health system, but need eventually to be replaced by trained nurses based at the health centre, after which peer educators confine themselves to community outreach and health promotion.

## Discussion and conclusions

Our case study aimed to analyse and evaluate the MoPoTsyo programme as an example of a patient-centred programme within the overall health system context. The results show how it is possible to create an enabling environment for self-management of patients with chronic diseases following an innovative and patient-centred approach. The evolution of this programme shows an adaptation in design, moving from an initiative initially focused on promoting diabetes self-management to a more comprehensive approach, which also comprises the removal of bottlenecks in access to medicines and services such as diagnostics and consultations. The programme succeeded in detecting essential barriers in the environment and the local health system hampering people’s access to appropriate care. The project managed to overcome these barriers through highly innovative solutions. Finally, the project was able to install a system of self-assessment and self-improvement of its own activities. In terms of integration, the project offers an example of how an initially vertical programme evolved to a comprehensive set of services while establishing links with the community, the public health system, private providers, and international organizations. Launched as a local initiative, the programme is now being scaled up within the national NCD strategy.

Our analyses further demonstrate how stakeholders perceive the multiple roles of MoPoTsyo in relation to their own position in the health system. The peer educator network is perceived as a competent network, relevant for community outreach and addressing patients’ demand. The peer educators themselves feel they respond to important community needs and some are ready to take up more tasks. Other health system stakeholders however, see peer educators as an extension to public health services and warn against relying too muchon peer educators’ skills and competence. Competition with other health providers was not considered problematic which suggests that this project does address an unanswered patient demand.

The RDF satisfies the needs of the beneficiaries and is essential for the efficiency and sustainability of the innovation, according to stakeholders. However, it is also radically different from the public procurement and supply of medicines in place and contributes to the major challenges of integration of the project into the public health system. Generally, there are concerns about sustainability and evolution of the model over time.

The collaboration with other health providers seems to depend on how visible the programme has been in the area, as reflected by the degree of activity of the peer educators. The government expresses a clear demand for a better collaboration with the public health system, while peer educators are cautious about the government taking over the initiative.

### Comparing with other research

Many case studies on peer educator initiatives elaborate on key functions, defined as assistance in self-management, social and emotional support and linkage to clinical care [10]. Peers are considered community-based supporters, encouraging patients to visit professional health services [35]. Pleas for more extensive roles of peer educators, come with the observation that most health care systems are not adapted to cope with this [36]. In a comparative study of peer programs in New Zealand, acceptance of peer educators was variable among professional staff, with some being supportive, others ready to replace them [13].

The innovation of task-shifting to peer educators is similar to strategies in other countries, even if the rationale and context in the MoPoTsyo case are somewhat different. In Sub-Saharan Africa, task-shifting was a response to a human resources crisis, when public health services failed to manage the workload created by HIV/AIDS in a situation where many health workers were themselves affected by the condition [37, 38]. In Cambodia, public health services were neglecting diabetes rather than facing overcrowding and a large demand. Also, task-shifting in Africa rather targeted lower qualified health workers or community health workers, than patients themselves [37, 39, 40]. From its conception, the MoPoTsyo vision has been to empower patients to take more control in the management of their disease; the initiative therefore goes a step further than the generally more instrumental vision of many other task-shifting strategies. As a result, the acceptability of the innovation among various stakeholder groups is also very variable.

### Limitations

The limitations of this study are related to its design, this being a case study approach, . While we aim to understand the dynamic nature of the implementation over time, the respondents’ view reflects the perception at the time of data collection. For instance, only the experience with private pharmacies, but not the later integration of the RDF into the public district hospitals was covered by stakeholder interviews. The important changes of the hand-over of some district-level projects to the Ministry of Health was not covered in the data collection. The interviews were conducted in a limited number of areas, which makes it difficult to generalize answers for the overall programme. The differences in answers also show the variation in the implementation of the programme. We were not able to include patients in this round of data collection. Previous evaluations covered patient perspectives about the programme [27].

Despite these limitations, we believe that our case study improves useful insights for understanding of the success and perceptions of patient-based approaches to chronic disease management in a health care system in an LMIC. By extending its focus from diabetes patients alone to include patients with hypertension, the MoPoTsyo programme shows that the programme principles can be applied to other chronic diseases. Important factors determining the outcomes in this case were: a consistent focus on and involvement of the target group backed up by a strong organization; a simultaneous reduction of other barriers to care; and maintaining good relations at all levels within the health care system.

To understand the MoPoTsyo position in the health care system, it is useful to take a market systems perspective [41]. MoPoTsyo was initiated to address a market failure that left diabetes patients without care. Peer educators, who are trained, trusted and available, have filled part of this gap. Over time, both the scale and scope of services have expanded, and the growing network of peer educators has become more tightly linked to other market players, such as pharmacies, laboratories, and public and private sector health service providers. These shifts in status have so far not been perceived as a serious threat to vested interests, but statements from government decision makers may indicate that some of the bottom-up initiatives may not be acceptable for scale-up in a public sector system.

Despite resistance among stakeholders in the field, the active role of people with chronic diseases in the management of their own disease is widely recognized as essential to improve the response of health care systems to the emergence of chronic diseases [42]. This requires the health care system managers and health service providers to change their way of thinking to empower the individual, rather than to control her/him, by using open communication respecting the individual as the central actor in her/his own care. The peer educators from MoPoTsyo are respected for their approach. Despite resistance of providers, MoPoTsyo has been successful in establishing a more balanced relationship between patients and health service providers, empowering patients to self-manage and demand services that meet their needs. People-centred health systems try to identify barriers to care and attempt to overcome them by creating the ideal circumstances for the individual to take care of him/herself [43].

## Abbreviations

NGO: Non-Governmental Organization
PE: peer educator
RDF: revolving drug fund

## References

[1] Mathers CD, Loncar D (2006). Projections of global mortality and burden of disease from 2002 to 2030. PLoS Med.

[2] World Health Organization (2010). Global status report on noncommunicable diseases.

[3] Marrero SL, Bloom DE, Adashi EY (2012). Noncommunicable diseases: a global health crisis in a new world order. JAMA.

[4] International Diabetes Federation (2014). IDF diabetes atlas.

[5] World Bank (2011). The growing danger of non-communicable diseases.

[6] Bloom DE, Cafiero ET, Jané-Llopis E, Abrahams-Gessel S, Bloom LR, Fathima S (2011). The global economic burden of Non-communicable diseases.

[7] Systems for Improved Access to Pharmaceuticals and Services (SIAPS) (2014). Enhancing health outcomes for chronic diseases in resource-limited settings by Improving the use of medicines: the role of pharmaceutical care.

[8] Ridaura RL, Wirtz VJ, Kaplan WA, Téllez YS-A (2011). Affordable, quality, long-term care and pharmacotherapy of chronic diseases: a framework for low and middle income countries.

[9] Heisler M (2007). Overview of peer support models to improve diabetes self management and clinical outcomes. Diabetes Spectr.

[10] Fisher EB, Earp JA, Maman S, Zolotor A (2010). Cross-cultural and international adaptation of peer support for diabetes management. Fam Pract.

[11] Funnell MM (2010). Peer-based behavioural strategies to improve chronic disease self-management and clinical outcomes : evidence, logistics , evaluation considerations and needs for future research. Fam Pract.

[12] Lorig K, Sobel DS, Ritter P, Laurent D, Hobbs M (2001). Effect of a self-management program on patients with chronic disease. Eff Clin Pract.

[13] Simmons D, Voyle J, Rush E, Dear M (2010). The New Zealand experience in peer support interventions among people with diabetes. Fam Pract.

[14] Werfalli M, Raubenheimer P, Engel M, Peer N, Kalula S, Kengne AP (2015). Effectiveness of community-based peer-led diabetes self-management programmes (COMP-DSMP) for improving clinical outcomes and quality of life of adults with diabetes in primary care settings in low and middle-income countries (LMIC): a systematic review. BMJ Open.

[15] Sheikh K, Gilson L, Agyepong IA, Hanson K, Ssengooba F (2011). Building the field of health policy and systems research : framing the questions. PLoS Med.

[16] Paina L, Peters DH. Understanding pathways for scaling up health services through the lens of complex adaptive systems. Health Policy Plan. 2012;27(5):365–73. doi:10.1093/heapol/czr054. 10.1093/heapol/czr054 21821667

[17] Plsek PE, Greenhalgh T (2001). Complexity science: the challenge of complexity in health care. BMJ.

[18] Crowe S, Cresswell K, Robertson A, Huby G, Avery A, Sheikh A (2011). The case study approach. BMC Med Res Methodol.

[19] International Diabetes Federation (2014). IDF diabetes atlas.

[20] United Nations Develment Programme (2013). Human development report 2013 The rise of the south: human progress in a diverse world.

[21] Grundy J, Khut QY, Oum S, Annear P, Ky V (2009). Health system strengthening in Cambodia-a case study of health policy response to social transition. Health Policy.

[22] Otgontuya D, Oum S, Buckley BS, Bonita R (2013). Assessment of total cardiovascular risk using WHO/ISH risk prediction charts in three low and middle income countries in Asia. BMC Public Health.

[23] Raguenaud M-E, Isaakidis P, Reid T, Chy S, Keuky L, Arellano G (2009). Treating 4,000 diabetic patients in Cambodia, a high-prevalence but resource-limited setting: a 5-year study. BMC Med.

[24] Isaakidis P, Raguenaud M, Say C, De Clerck H, Khim C, Pottier R (2011). Treatment of hypertension in rural Cambodia: results from a 6-year programme. J Hum Hypertens.

[25] Thomas B, Van Pelt M, Mehrotra R, Robinson-Cohen C (2013). An estimate of reduced eGFR in a rural cambodian diabetic population.

[26] Van Olmen J, Marie KG, Christian D, Clovis KJ, Emery B, Maurits VP, et al. Content, participants and outcomes of three diabetes care programmes in three low and middle income countries. Prim Care Diabetes. 2015;9(3):196–202. doi:10.1016/j.pcd.2014.09.001. 10.1016/j.pcd.2014.09.001 25281167

[27] Eggermont N (2011). Evaluation of a peer-education program for diabetes and hypertension in rural cambodia.

[28] Eggermont N, Van Olmen J, Van Pelt M, Van Damme W (2014). Alliance for health policy and systems research flagship report 2014. Chapter 5 - Annex 2. Task-shifting to peer educators in Cambodia.

[29] Elo S, Kyngäs H (2008). The qualitative content analysis process. J Adv Nurs.

[30] Van Pelt M (2010). Peer educator networks for people with diabetes mellitus or high blood pressure in Cambodia. ‘Healing a market for health.’ 2005–2010,” MoPoTsyo.

[31] Men C (2007). ‘I Wish I Had AIDS’ Qualitative study on health care access among HIV / AIDS and diabetic patients in Cambodia.

[32] MoPoTsyo (2013). Annual report 2012.

[33] Alberti KP, Guthmann JP, Fermon F, Nargaye KD, Grais RF (2008). Use of Lot Quality Assurance Sampling (LQAS) to estimate vaccination coverage helps guide future vaccination efforts. Trans R Soc Trop Med Hyg.

[34] Ministry of Health Cambodia (2013). Strategic plan for the prevention of NCD 2013–2020.

[35] Fisher EB, Bootroyd I, Coufal M, Bauman L, Mbanya J, Rotheram-Borus MJ (2012). Peer support for self-management of diabetes improved outcomes in international settings. Health Aff.

[36] Baksi AK (2010). Experiences in peer-to-peer training in diabetes mellitus: challenges and implications. Fam Pract.

[37] Fulton BD, Scheffler RM, Sparkes SP, Auh EY, Vujicic M, Soucat A (2011). Health workforce skill mix and task shifting in low income countries: a review of recent evidence. Hum Resour Health.

[38] Kober K, Van Damme W. Expert patients and AIDS care: A literature review on expert patient programmes in high-income countries, and an exploration of their relevance for HIV/AIDS care in low-income countries with severe human resource shortages. Antwerp. 2006. http://www.equinetafrica.org/bibl/docs/KOBaids.pdf

[39] Lekoubou A, Awah P, Fezeu L, Sobngwi E, Kengne AP (2010). Hypertension, diabetes mellitus and task shifting in their management in sub-Saharan Africa. Int J Environ Res Public Health.

[40] Labhardt ND, Balo J-R, Ndam M, Grimm J-J, Manga E (2010). Task shifting to non-physician clinicians for integrated management of hypertension and diabetes in rural Cameroon: a programme assessment at two years. BMC Health Serv Res.

[41] Bloom G, Kanjilal B, Lucas H, Peters D (2012). Transforming health markets in Asia and Africa: improving quality and access for the poor.

[42] Atun R, Jaffar S, Nishtar S, Knaul FM, Barreto ML, Nyirenda M (2013). Improving responsiveness of health systems to non-communicable diseases. Lancet.

[43] Sheikh K, Ranson MK, Gilson L (2014). Explorations on people centredness in health systems. Health Policy Plan.

